# Comparison of Growth Performance and Plasma Metabolomics between Two Sire-Breeds of Pigs in China

**DOI:** 10.3390/genes14091706

**Published:** 2023-08-27

**Authors:** Zhongwei Xie, Mailin Gan, Junhua Du, Gao Du, Yi Luo, Bin Liu, Kangping Zhu, Wenqiang Cheng, Lei Chen, Ye Zhao, Lili Niu, Yan Wang, Jingyong Wang, Li Zhu, Linyuan Shen

**Affiliations:** 1 Farm Animal Genetic Resources Exploration and Innovation Key Laboratory of Sichuan Province, Sichuan Agricultural University, Chengdu 611130, China; xiezhongwei@stu.sicau.edu.cn (Z.X.); ganmailin@sicau.edu.cn (M.G.);; 2 Key Laboratory of Livestock and Poultry Multi-Omics, Ministry of Agriculture and Rural Affairs, College of Animal and Technology, Sichuan Agricultural University, Chengdu 611130, China; 3 Sichuan Dekon Livestock Foodstuff Group, Chengdu 610200, China; 4 National Animal Husbandry Service, Beijing 100125, China; 5 Chongqing Academy of Animal Science, Chongqing 402460, China

**Keywords:** Duroc, cross system, paternal Yorkshire pig, plasma metabolomics

## Abstract

The Yorkshire pigs, renowned for their remarkable growth rate, low feed conversion ratio (FCR), and high meat production, emerge as a novel preference for paternal breeding. In this study, we found that purebred paternal Yorkshire pigs (PY) surpass the purebred Duroc breed in terms of growth rate. Specifically, purebred PY attain a weight of 100 kg at an earlier age compared to purebred Duroc (Male, 145.07 vs. 162.91; Female, 145.91 vs. 167.57; *p*-value < 0.01). Furthermore, different hybrid combinations suggest that offspring involving purebred PY exhibit superior growth performance. Compared with purebred Duroc, the offspring of purebred PY have an earlier age in days (173.23 vs. 183.54; *p*-value < 0.05) at the same slaughter weight. The changes of plasma metabolites of 60-day-old purebred boars in the two sire-breeds showed that 1335 metabolites in plasma were detected. Compared with Duroc, 28 metabolites were down-regulated and 49 metabolites were up-regulated in PY. Principal component analysis (PCA) discerned notable dissimilarities in plasma metabolites between the two sire-breeds of pigs. The levels of glycerol 3-phosphate choline, cytidine, guanine, and arachidonic acid increased significantly (*p*-value < 0.05), exerting an impact on their growth and development. According to our results, PY could be a new paternal option as a terminal sire in three-way cross system.

## 1. Introduction

Specialized sire and dam line pigs are cultivated in modern pig breeding systems, with sire lines bred for excellent growth performance and high lean meat percentage, and dam lines emphasizing reproductive performance and maternal behavior. China is the unparalleled global leader in swine breeding, pork production, and consumption, having a substantial influence on worldwide markets [1,2]. Duroc has emerged as the predominant option for specialized paternal pig breeding in China [3], primarily due to its rapid growth rate, elevated slaughter rate, high lean meat percentage, and promising economic returns [4]. Therefore, 70% or more of pork production in China comes from DLY pigs, which are produced by hybrid breeding Duroc with (Landrace × Yorkshire) pigs [5]. Nevertheless, in recent years, some weaknesses for Duroc have been identified, i.e., it exhibits low heritability of feed efficiency, a limited number of progenies, and a tendency to lose weight under abnormal conditions [6,7]. Accordingly, more and more commercial breeding companies hope to develop different paternal pig breeds. Compared with Duroc, Yorkshire pigs not only have a faster growth rate and higher lean meat rate, but also have their bodybuilding appearance, excellent meat production performance and good stress resistance [8,9]. Commercial breeding companies have successively cultivated new strains of paternal Yorkshire pigs according to market demand differences. For example, Topigs Norsvin (TN) in Dutch has used Large White pigs as terminal sires to develop the TN Tempo swine, renowned for their robust piglet vitality, sturdy limb muscles, remarkable fattening performance, and high feed conversion rate. 

The metabolome may be conceptualized as comprising four principal biochemical domains: protein metabolism, carbohydrate metabolism, nucleic acid metabolism, and fatty acid metabolism [10]. A comprehensive examination of the pig metabolome yields profound insights into the intricate molecular underpinnings of physiological functionality, growth and development [11], genetic improvement, feed nutrition [12], meat quality [13], reproductive function [14,15], stress response [16], and even the identification of maladies [17]. The focus is, on the one hand, to find reliable biomarkers of animal phenotypic characteristics [18], and, on the other hand, to analyze the material basis behind the different phenotypes [19]. Previous studies have confirmed plasma/serum metabolome, which more closely captures the host’s metabolism, can differentiate between two pig breeds [20]. Similarly, Rohart et al. used plasma metabolite detection results from different breeds to show that amino acid metabolite indicators such as lactate, alanine, isoleucine, and valine are related to the lean meat rate of different breeds of pigs [21]. The research on pigs has demonstrated the advantages of metabolomics, providing new materials for the screening and rapid and accurate identification of biomarkers related to economic traits in pigs, and providing important references for understanding and recognizing the formation of important phenotypic traits in pigs.

Therefore, in this study, we compared the growth performance of purebred Duroc and purebred PY, and also used hybrid systems (‘DLY’ and ‘YLY’) to compare the growth rates of their hybrid offspring. Finally, we conducted a plasma metabolomic analysis of 60-day-old purebred Duroc boars and purebred PY boars.

## 2. Materials and Methods

### 2.1. Ethics Approval Statement

All experimental procedures described below were approved by the Animal Ethical and Welfare Committee of Sichuan Agricultural University, Chengdu, China (Approval No. 2021302137, approval date: 1 July 2021). 

### 2.2. Animals and Sample Collection

The experiment used animals sourced from a pig breeding company located in Sichuan Province, China, which were reared under identical conditions. All pigs had unrestricted access to the same feeding regimen and water supply. The growth data of the hybrid offspring of ‘DLY’ and ‘YLY’ comes from six different pig farms of the same company. The plasma samples originated from a cohort of pigs raised in a uniform and homogenous environment to ensure experimental controls.

### 2.3. Determination of Growth Performance

Backfat thickness and eye-muscle area were measured using a B-mode real-time ultrasound. Body size indices included body weight (BW), body length (BL), body height (BH), chest circumference (CC), abdominal circumference (AC), hip circumference (HC), chest width (CW), and chest depth (CD).

### 2.4. Plasma Sample Preparation for LC-MS/MS

The plasma metabolomics study was conducted on 10 pigs of two different breeds: 5 male PY and 5 male Duroc (60 days of age). The anterior vena cava blood collection method was used to collect 5 mL of venous blood, which was then placed in an anticoagulant tube containing EDTA (ethylene-diamine-tetraacetic-acid). After standing at room temperature for 30 min, the samples were centrifuged at 3000 rpm/min for 10 min, divided in several aliquots, and stored at −80 °C till LC-MS/MS analysis. The 100 μL samples were placed within the Eppendorf (EP) tubes and reconstituted with prechilled 80% methanol using a robust vortexing technique. Subsequently, the samples were incubated on ice for 5 min and then subjected to centrifugation at 15,000× *g* and 4 °C for a duration of 20 min. A portion of the resulting supernatant was appropriately diluted to achieve a final concentration comprising 53% methanol, with the addition of LC-MS grade water. Following this, the samples were meticulously transferred to a fresh EP tube and once again subjected to centrifugation at 15,000× *g* and 4 °C for another 20 min. Finally, the supernatant was injected into the LC-MS/MS system for comprehensive analysis. Quality control (QC) samples were equal volume mixed samples from experimental samples. They served the purpose of harmonizing the chromatography-mass spectrometry system, monitoring instrument performance, and assessing system stability throughout the entirety of the experimental procedure. Simultaneously, the incorporation of blank samples primarily aimed to eliminate background ions.

### 2.5. Instruments and Settings

LC-MS/MS analyses were conducted employing a Vanquish LC-MS/MS system (ThermoFisher, Karlsruhe, Germany). The samples were injected onto a Hypesil Goldcolumn (100 × 2.1 mm, 1.9 μm) using a precise 17-min linear gradient at a flow rate of 0.2 mL/min. For the positive polarity mode, eluent A (0.1% FA in Water) and eluent B (Methanol) were employed, while for the negative polarity mode, eluent A (5 mM ammonium acetate, pH 9.0) and eluent B (Methanol) were used. The solvent gradient was set as follows: 2% B for 1.5 min, followed by a rapid increase to 100% B over 3 min, maintaining 100% B for 10 min, then decreasing to 2% B over 10.1 min, and finally reaching a steady state at 2% B for an additional 12 min. The Q ExactiveTM HF mass spectrometer was operated in both positive and negative polarity modes, with a spray voltage of 3.5 kV, a capillary temperature of 320 °C, a sheath gas flow rate of 35 psi, an auxiliary gas flow rate of 10 L/min, an S-lens RF level of 60, and an auxiliary gas heater temperature of 350 °C. 

### 2.6. Data Processing and Metabolite Identification

The raw data files generated by LC-MS/MS underwent processing using the sophisticated Compound Discoverer 3.1 (CD3.1, ThermoFisher) to conduct peak alignment, peak picking, and quantitation for each metabolite. The main parameters were set as follows: retention time tolerance of 0.2 min, actual mass tolerance of 5 ppm, signal intensity tolerance of 30%, signal/noise ratio of 3, and a minimum intensity threshold. Subsequently, the peak intensities were normalized to the total spectral intensity. The normalized data were employed to predict the molecular formula based on additive ions, molecular ion peaks, and fragment ions. Following this, the peaks were meticulously matched with the mzCloud (https://www.mzcloud.org/, accessed on 25 August 2022), mzVault, and MassList databases to obtain accurate qualitative and relative quantitative results. For statistical analyses, the software tools R (R version R-3.4.3), Python (Python 2.7.6 version), and CentOS (CentOS release 6.6) were utilized. 

### 2.7. Data Analysis

The preprocessing yielded a data matrix comprising the retention time (RT), mass-to-charge ratio (*m*/*z*) values, and peak intensity. These metabolites were annotated, and pathway analysis was performed on the differential metabolites via the LIPIDMaps (http://www.lipidmaps.org/, accessed on 25 August 2022), HMDB (https://hmdb.ca/metabolites, accessed on 25 August 2022), and KEGG (https://www.genome.jp/kegg/pathway.html, accessed on 25 August 2022) databases. Principal components analysis (PCA) and partial least squares discriminant analysis (PLS-DA) were performed at metaX. We utilized univariate analysis (*t*-test) to compute the statistical significance (*p*-value). Unless otherwise indicated, the criteria for screening differential metabolites are VIP score > 1, *p*-value < 0.05, and |FC| ≥ 2. 

Regarding statistical analysis, WPS Office was used to enter and preliminarily collate data. Statistical analysis was conducted using SPSS 22.0 (IBM, USA) through one-way ANOVA for performance measurements of purebred pigs of different genders and correlation analysis of each index. We used a student’s *t*-test to analyze the sales situation of ‘YLY’ and ‘DLY’ offspring. The results were generally expressed as mean ± standard deviation, unless otherwise noted. The results of correlation were interpreted according to statistical significance as follows: *p* > 0.8 (strong correlation), 0.5 ≤ *p* ≤ 0.8 (moderate correlation), *p* ≤ 0.5 (weakly correlated).

## 3. Results

### 3.1. Determination of Growth Performances of Purebred PY and Purebred Duroc

Sire-breeds are used to improve the growth rate and meat quantity of hybrid-breed pig lines [22]. Therefore, the age of days to 100 kg, backfat thickness, and the eye-muscle area of purebred PY and Duroc, born on similar dates and raised in the same farm, were measured (Table 1). For the age of days to 100 kg, male and female PY were markedly inferior to that of Duroc (female, 145.91 vs. 167.57; male, 145.07 vs. 162.91; *p*-value < 0.01), with no statistically significant variance discerned between the genders. Meanwhile, the area of the eye-muscle of PY were significantly larger than Duroc pigs (Table 1). In relative terms, pigs with thinner backfat thickness and larger eye-muscle area have a higher lean meat rate. 

**Figure 1:**
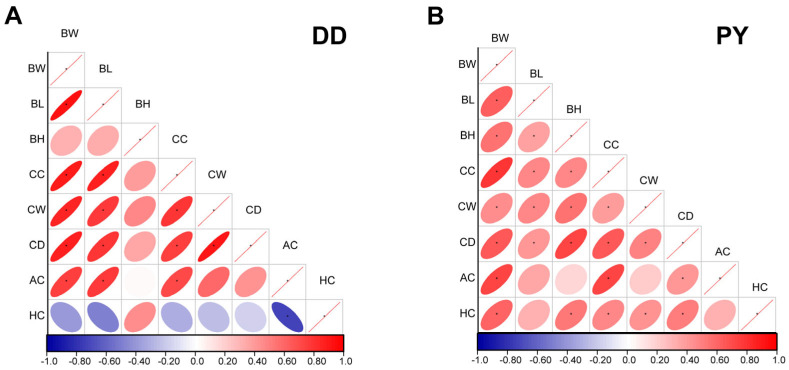
Pearson correlation coefficient of body size data of purebred Duroc (**A**) and PY (**B**). BW (body weight); BL (body length); BH (body height); CC (chest circumference); CW (chest width); CD (chest depth); AC (abdominal circumference); HC (hip circumference). A positive correlation is denoted by the color red, while a negative correlation is represented by the color blue. The size of each circle corresponds to the correlation coefficient, whereby larger circles indicate a higher correlation coefficient. * represents *p*-value < 0.05, which indicated significant difference.

To explore the reasons for weight differences, we further conducted Pearson correlation analysis between weight, age, and body size data. The body weight of PY showed a moderate positive correlation with all indicators, while the body weight (BW) of Duroc was highly positively correlated with body length (BL), chest circumference (CC), chest width (CW), and chest depth (CD), but negatively correlated with hip circumference (HC) (Figure 1A,B).

### 3.2. Growth Performance of Hybrid Offspring

Based on the above results, PY have a faster growth rate and higher lean meat percentage but no difference in body size data compared to Duroc, with regards to sire-breed. As such, we tracked and analyzed the fattening performance of ‘YLY’ (PY × (Landrace × Yorkshire)) and ‘DLY’ (Duroc × (Landrace × Yorkshire) hybrid pigs. The results showed that, on the three pig farms that bred ‘YLY’ hybrid pigs, the feeding days were earlier than those of the farms raising ‘DLY’ hybrid pigs. Additionally, ‘YLY’ hybrid pigs had a fattening period that was 11.31 days lower than that of ‘DLY’ hybrid pigs and the selling weight only differed by 1.03 kg. Furthermore, the average daily feed intake of ‘YLY’ hybrid pigs was higher (104.41 g) than that of ‘DLY’ hybrid pigs, and the feed weight ratio exhibited a decrease of 0.004. On average, the fattening period of ‘YLY’ hybrid pigs was shortened by 10.31 days, while the difference in sales weight was less than 0.1 kg. Finally, ‘YLY’ hybrid pigs recorded an average daily feed intake that was 89.65 g higher than that of ‘DLY’ hybrid pigs, and the feed conversion ratio observed a decrease of 0.03. Overall, the observed advantages of ‘YLY’ hybrid pigs in our experimental conditions included a shortened fattening period by about 10 days while reaching a market weight of about 136 kg (Table 2).

### 3.3. Analysis of Plasma Differential Metabolites between Duroc and PY

**Figure 2:**
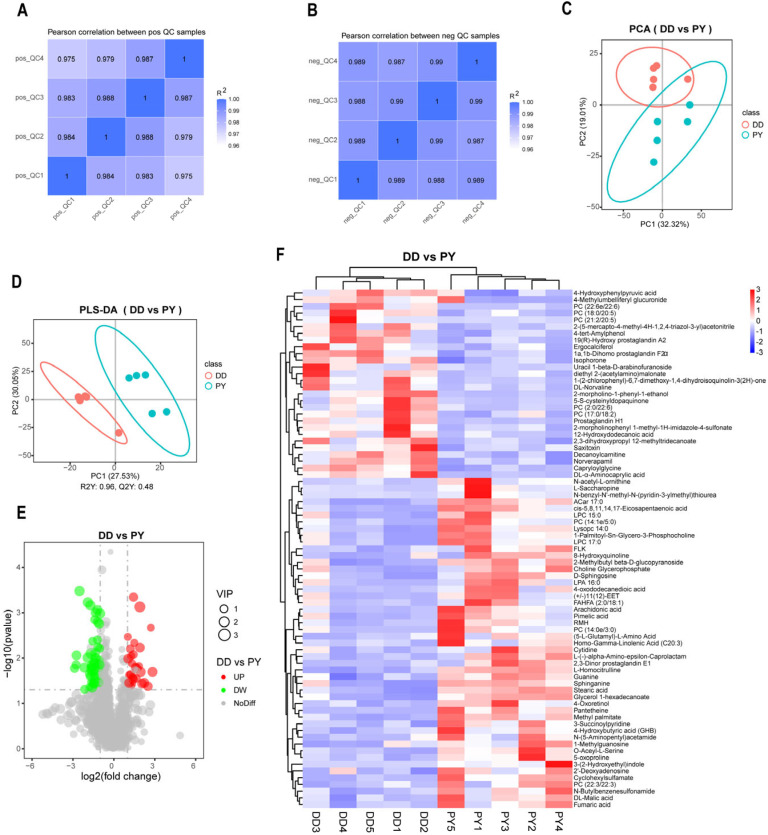
Analysis of plasma differential metabolites between PY and Duroc. Pearson correlation coefficients of four quality control (QC) samples in the positive-ion mode (**A**) and the negative-ion mode (**B**). (**C**) The PCA score plot of metabolite data. (**D**) The PLS-DA score plot of metabolite data. (**E**) Differential metabolite volcano diagram. (**F**) Differential metabolite clustering heatmap.

PY showed excellent growth performance both in purebred pigs and their hybrid offspring, and in order to further analyze this difference, we selected the boars at the beginning of fattening. The plasma metabolic profiles of male Duroc and male PY purebred pigs were detected using untargeted metabolomics techniques. Firstly, we assessed the correlation of QC samples in positive and negative ions using Pearson correlation coefficients (Figure 2A,B). The correlations of all four QC samples in this study were higher than 0.975, indicating good stability of the detection process and reliable measurement results. A total of 1335 metabolites (883 metabolites in positive ions pattern and 452 metabolites in negative ions pattern) in plasma were detected. The PCA showed that the plasma metabolite patterns of PY and Duroc were different, and the plasma metabolites of Duroc had less variation among samples (Figure 2C). The PLS-DA model further distinguished the difference between the PY and DD groups (Figure 2D). The plasma metabolite patterns of Duroc and PY were different, which can be well distinguished into two categories. The results of the PCA and PLS-DA indicated significant differences in plasma metabolites between PY and Duroc. Further differential analysis of the detected plasma metabolites revealed that 49 metabolites were down-regulated and 28 metabolites were up-regulated in Duroc compared with PY (Figure 2E). Among the metabolites with KEGG_ID identified by differential metabolic species, 22 were down-regulated and 6 were up-regulated (Supplementary Table S1). Heatmaps of differential metabolites varying between pig breeds evidenced that the plasma samples of Duroc and PY can be separated independently. In addition, differential metabolites can be well clustered into up-regulated and down-regulated classes (Figure 2F).

### 3.4. Correlation and Functional Enrichment Analysis of Differential Metabolites

**Figure 3:**
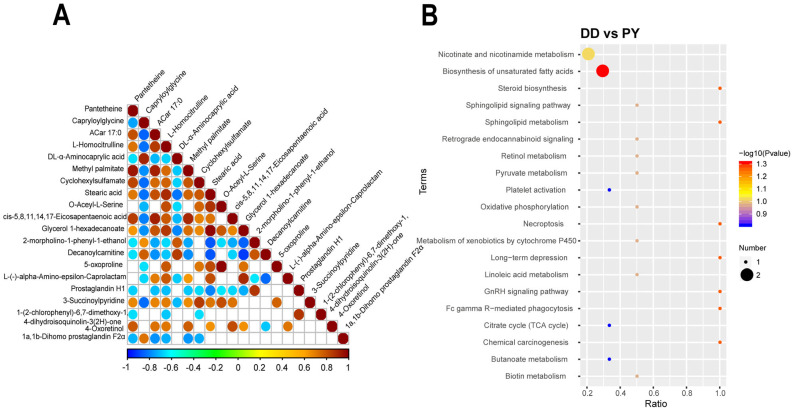
The correlation of differential metabolites is represented by blue and red, indicating negative and positive correlations, respectively (**A**). The KEGG enrichment bubble diagram of differential metabolites (**B**).

The above results show that the Duroc pig plasma samples and PY plasma samples had good intra-group repeatability and large inter-group differences. The correlation of differential metabolites were evaluated (Figure 3A), and were mainly moderately negative correlated. We performed functional annotation and metabolic pathway enrichment analysis of differential plasma metabolites by the KEGG database, and the results suggested that these differential metabolites were mainly enriched in nicotinate and nicotinamide metabolism, biosynthesis of unsaturated fatty acids metabolic pathways, and these pathways are both involved in metabolism. The metabolites involved in the biosynthesis of unsaturated fatty acids metabolic pathways include arachidonic acid, and stearic acid. The enrichment analysis showed that differential metabolites were mainly involved in biosynthesis of flavonoids, amino acids, alkaloids, etc. (Figure 3B).

## 4. Discussion

In previous breeding practices, Duroc boars were widely employed as terminal male parents in three-way hybrid systems due to their rapid growth, substantial feed rewards, excellent slaughter rates, carcass traits, and meat quality traits. However, the dwindling number of Duroc boars, along with their hybrid offspring’s subpar reproductive traits, slow growth and development in harsh breeding environments, and poor stress resistance, has become a concern in recent years. Consequently, international breeding companies are developing new breeds with comparable traits to Duroc boars as terminal male parents in response to market demand (for example, paternal Yorkshire pigs). This study’s primary objective was to contrast the growth performance and body size of Duroc and PY, and the growth rate of hybrid offspring between PY and traditional terminal paternal Duroc and to explore the reasons for the variations between the two sire-breeds from plasma metabolomics. 

At present, Duroc are primarily utilized in breeding programs as paternal lines, whereas hybrids between Landrace and Yorkshire pigs are employed as maternal lines [22]. ‘DLY’ (Duroc × (Landrace × Yorkshire)) is the hybrid breed of a three-way hybrid system [23], which was the most mature commercial hybrid matching system and the most dominant hybrid breeding system. Based on the above research, we further carried out the hybrid utilization of PY and Duroc sires. In this experiment, the main advantage of ‘YLY’ (PY × (Landrace × Yorkshire)) hybrid pigs was effectively improved growth performance of finishing pigs, indicating that ‘YLY’ may shorten by about 10 days the duration of the fattening period to allow pigs to reach slaughter weight of about 136 kg. In the operation of breeding companies, the same market weight for pigs, the smaller the age of the pig, the lower the risk and the more substantial the profit for the company. 

Specific genetic backgrounds are important factors affecting metabolic status. Studies have shown that the heritability of long-chain fatty acid metabolism molecules in milk is between 0.05 and 0.38 [24], and the heritability of milk protein is between 0.05 and 0.78 [25]. Studies on pigs have found that the heritability of long-chain polyunsaturated fatty acids in pork is above 0.50 [26], and the heritability of lipid metabolism molecules in the backfat of Iberian pig ranges from 0.06 to 0.53 [27]. There are differences in liver and blood metabolites among different pig breeds and are associated with meat quality indicators [28]. To date, there is no relevant article directly expounding the relationship between plasma metabolites and breeding traits in pigs, but there were also many articles reporting that plasma metabolites were associated with related indicators such as body weight [29], growth rate [30,31], fat deposition [20], and reproductive performance [32]. There have been reports suggesting a positive correlation between sphingomyelin and fat deposition [33]. Bovo found that the plasma metabolites of sphingomyelin were significantly higher in Italian Duroc pigs compared to Italian Large White pigs [20]. This is consistent with reports that Italian Duroc pigs have higher intramuscular fat deposition than Italian Large White pigs. Previous studies have demonstrated that acetoacetate acid, triacylglycerols, phosphatidylcholine, specific amino acids, as well as creatine and creatinine in plasma metabolites, can serve as reliable indicators for predicting weight loss [29]. Analysis of plasma metabolites in lambs from the same pasture showed a significant positive correlation between growth rate and at least five plasma metabolites, including the amino acids valine, methionine, cystine asparagine, and phosphoric acid [31]. 

The plasma metabolic profiles of PY and Duroc were detected using untargeted metabolomics techniques in this study. Previous studies based on differences in body fluid metabolites between Italian Large White pigs and Duroc confirmed that genetic differences are some of the main factors for differences in body fluid metabolites [20]. A total of 1335 metabolites (883 metabolites in positive ions pattern and 452 metabolites in negative ions pattern) in plasma were detected in this study. Further differential analysis of the detected plasma metabolites revealed that 49 metabolites were down-regulated and 28 metabolites were up-regulated in Duroc compared with PY. Up-regulated differentially metabolized species in Duroc can be annotated to KEGG_ID, such as: Saxitoxin, 4-Hydroxyphenylpyruvic acid, 12-Hydroxydodecanoic acid, Ergocalciferol, isophorone, and DL-Norvaline. Ergocalciferol (Vitamin D2) and cholecalciferol (vitamin D3) are the most common forms of vitamin D [34]. 4-hydroxyphenylpyruvic acid (4-HPPA) is a keto acid that plays a crucial role in the catabolic pathway of tyrosine [35]. In particular, 4HPPA can be biosynthesized from L-tyrosine through its interaction with tyrosine aminotransferase. Vitamin C, essential in the oxidative degradation of tyrosine, exhibits a close association with the activity of HPPD. This indicates that 4-HPPA holds promise as a valuable marker for gauging vitamin C bioavailability and uptake [35]. In this study, a noteworthy reduction in 4-hydroxyphenylpyruvic acid (4-HPPA) was observed in Duroc, implying an aberrant metabolism of tyrosine and a potential link to vitamin C bioavailability. Studies have shown that increasing Ergocalciferol significantly reduces the expression of adipose genes, thereby reducing adipose tissue expansion and preventing obesity [36]. Up-regulated metabolic species in PY can be annotated to KEGG_ID as Pantetheine, [37] and choline glycerophosphate [38], which have been shown to contribute to fat deposition. In addition, guanine, cytidine, and 2′-Deoxyadenosine are involved in cell metabolism and nucleic acid synthesis [39,40]. Choline glycerophosphate, comprising choline, glycerol, and phosphate [41], serves as the choline source essential for the synthesis of acetylcholine, a neurotransmitter directly linked to body weight [42]. These results are consistent with the law of differences in growth performance between Duroc and PY. 

Cluster analysis can separate Duroc and PY well, indicating that the repeatability within the group is good. The results of KEGG suggested that the differential metabolites of Duroc and PY were mainly enriched in nicotinate and nicotinamide metabolism, which is the amide form of niacin, also known as vitamin B3, an essential vitamin [43], and biosynthesis of unsaturated fatty acids metabolic pathways. Nicotinate and nicotinamide metabolism signaling pathway is related to energy metabolism, lipid metabolism, and protein metabolism [44], while unsaturated fatty acids are mainly related to the metabolism of the body and lipid metabolism [45]. Arachidonic acid, which is involved in the biosynthesis of unsaturated fatty acids metabolic pathways stands as a proinflammatory indicator entwined with glucose metabolism and the regulation of body weight [46].

## 5. Conclusions

With the increasing commercialization of breeding, the development of better specialized strains has become a trend. In this study, we show that PY have better growth performances and higher lean meat percentage than Duroc. Moreover, for hybrid-breeds, we also found that the growth rate of PY offspring is better than that of Duroc. Furthermore, we found difference in plasma metabolites between the two breeds, such as significant up-regulation of choline glycerophosphate, cytidine, guanine, and arachidonic acid in PY, which may in turn affect the growth and development of pigs. In conclusion, PY may be better suited as terminal male parents in the breeding system than Duroc. Meanwhile, PY provides prospects for the growth potential and meat quality improvement of pigs, thereby contributing to the optimization of pig breeding strategies. By unraveling these pivotal insights, we offer sustainable and innovative approaches in the realm of swine production.

## Figures and Tables

**Table 1:** Performance measurement of purebred PY and Duroc.

Sex	Breed	Age of Days to 100 kg, Days	Average backfat thickness, mm	Eye-muscle area, cm^2^
Male	PY (*n* = 15)	145.07 ± 2.58 **	7.69 ± 1.48	67.77 ± 3.82 **
	Duroc (*n* = 12)	162.91 ± 6.73	7.6 ± 2.15	55.93 ± 7.14
Female	PY (*n* = 15)	145.91 ± 1.51 **	7.54 ± 1.33	64.75 ± 4.78 **
	Duroc (*n* = 12)	167.57 ± 7.96	8.58 ± 1.73	55.07 ± 5.44

Note: The performance measurement data sourced from purebred PY and Duroc (PY, male/female, n = 15; Duroc, male/female, n = 12). ** represents *p*-value < 0.01, which indicated significant difference.

**Table 2:** Fattening performance of ‘DLY’ and ‘YLY’ hybrid offspring.

Group	Numbers	Days	Weight, kg	Average Daily Feed Intake, g	Feed Conversion Ratio
‘YLY’ 1	1660	168.97	129.62	1942.43	2.64
‘YLY’ 2	1141	176.67	136.31	1867.80	2.53
‘YLY’ 3	1018	174.05	144.86	1994.52	2.49
‘DLY’ 1	1057	183.84	139.35	1831.23	2.53
‘DLY’ 2	995	179.82	136.38	1996.62	2.76
‘DLY’ 3	1380	186.96	134.93	1707.96	2.47
‘YLY’ #	3819	173.23 ± 3.91 **	136.93 ± 7.64	1934.92 ± 63.69	2.55 ± 0.08
‘DLY’ #	3432	183.54 ± 3.58	136.89 ± 2.25	1845.27 ± 144.84	2.58 ± 0.15

‘YLY’ 1, ‘YLY’ 2, and ‘YLY’ 3 refer to different fattening farms, respectively, and ‘DLY’ is the same. # is the average analysis based on different farms as the basic unit. Both pigs were weaned for 21 days, and the feeding conditions and feed were the same. ** represents *p*-value < 0.01, which indicated significant difference.

## Data Availability

The data presented in this study can be obtained directly from the corresponding authors upon request. Due to the size of the dataset, it is not currently available for public access.

## Supplementary Materials

The following supporting information can be downloaded at: https://www.mdpi.com/article/10.3390/genes14091706/s1, Table S1: Partial differential plasma metabolites in Duroc and PY (DD vs. PY).

## Abbreviations

AC	Abdominal circumference
BH	Body height
BL	Body length
BW	Body weight,
CC	Chest circumference
CD	Chest depth
CW	Chest width
DLY	Duroc × (Landrace × Yorkshire)
FCR	Feed Conversion Ratio
HC	Hip circumference
PCA	Principal component analysis
PY	paternal Yorkshire pigs
QC	Quality Control
TN	Topigs Norsvin
YLY	paternal Yorkshire × (Landrace × Yorkshire)
