# Supplementary material for: Comparison of Growth Performance and Plasma Metabolomics between Two Sire-Breeds of Pigs in China
Source: Genes (Basel). 2023 Aug 27;14(9):1706. doi: 10.3390/genes14091706 (PMC10531030; doi:10.3390/genes14091706)
Supplement: Supplementary file 1 [file genes-14-01706-s001.zip › genes-2554503-supplementary.pdf]

Table S1 Partial differential plasma metabolites in PY and Duroc (DD vs PY).

Order Number	Name	Molecular Weight	KEGG_ID	FC	*p*-value	VIP
1	3-(2-Hydroxyethyl) indole	161.08	C00955	0.15	0.01	2.14
2	Pantetheine	278.13	C00831	0.17	0.00	2.49
3	(+/-)11(12)-EET	320.24	C14770	0.22	0.02	2.33
4	L-Saccharopine	276.13	C00449	0.29	0.04	1.24
5	1-Palmitoyl-Sn-Glycero-3-Phosphocholine	495.33	C04230	0.30	0.02	2.40
6	L-Homocitrulline	189.11	C02427	0.30	0.00	2.38
7	Sphinganine	301.30	C00836	0.37	0.01	2.92
8	1-Methylguanosine	297.11	C04545	0.38	0.02	1.33
9	Choline Glycerophosphate	257.10	C00670	0.38	0.02	2.10
10	Arachidonic acid	304.24	C00219	0.39	0.01	2.20
11	Cyclohexylsulfamate	179.06	C02824	0.40	0.00	2.22
12	5-oxoproline	129.04	C01879	0.43	0.00	1.53
13	3-Succinoylpyridine	179.06	C19569	0.44	0.01	1.50
14	cis-5,8,11,14,17-Eicosapentaenoic acid	302.22	C06428	0.44	0.00	2.21
15	Cytidine	243.09	C00475	0.47	0.03	1.96
16	Fumaric acid	116.01	C00122	0.48	0.02	1.66
17	Pimelic acid	114.07	C02656	0.48	0.02	1.73
18	DL-Malic acid	134.02	C03668	0.48	0.01	1.68
19	4-Oxoretinol	300.21	C16683	0.49	0.01	1.57
20	Guanine	151.05	C00242	0.49	0.01	2.14

Order Number	Name	Molecular Weight	KEGG_ID	FC	*p*-value	VIP
21	2'-Deoxyadenosine	273.08	C00559	0.49	0.03	1.08
22	Stearic acid	284.27	C01530	0.50	0.00	2.43
23	Saxitoxin	281.12	C13757	2.04	0.03	1.10
24	4-Hydroxyphenylpyruvic acid	180.04	C01179	2.17	0.04	2.14
25	12-Hydroxydodecanoic acid	216.17	C08317	2.21	0.02	1.41
26	Ergocalciferol	396.34	C05441	2.35	0.02	1.60
27	Isophorone	138.10	C14743	2.52	0.01	1.25
28	DL-Norvaline	117.08	C01799	2.67	0.03	1.93

Metabolites with KEGG_ID are shown in this table, and those without KEGG_ID are not marked. VIP > 1.0, |FC| > 2, p-value < 0.05.
